# Combined laparoscopic and thoracoscopic surgical repair of Bochdalek hernia in an adult: a case report

**DOI:** 10.1186/s40792-021-01294-5

**Published:** 2021-09-21

**Authors:** Sho Nambara, Yoshihisa Sakaguchi, Fumihiro Shoji, Yasuo Tsuda, Kensuke Kudou, Eiji Kusumoto, Kenkichi Hashimoto, Tetsuya Kusumoto, Koji Ikejiri

**Affiliations:** 1 Department of Gastroenterological Surgery and Clinical Research Institute Cancer Research Division, National Kyushu Medical Center, 1-8-1 Jigyouhama Chuo-ku, Fukuoka, 810-8563 Japan; 2 Department of Thoracic Surgery, National Kyushu Medical Center, 1-8-1 Jigyohama Chuo-ku, Fukuoka, 810-8563 Japan

**Keywords:** Bochdalek hernia, Laparoscopy, Thoracoscopy

## Abstract

**Background:**

Bochdalek hernia is a rare disease in adults. Diaphragmatic hernia in adults has been repaired using minimally invasive surgery through laparoscopy or thoracoscopy. However, the literature regarding the combined use of laparoscopy and thoracoscopy for the repair of Bochdalek hernia is limited.

**Case presentation:**

A 26-year-old man diagnosed with Bochdalek hernia was managed through combined abdominal and thoracic endoscopic surgery. On laparoscopy, the omentum prolapsed into the left thoracic cavity through the posterolateral area of the left diaphragm. On thoracoscopy, no adhesions of the omentum were seen in the thoracic cavity. The omentum was drawn back to the abdominal cavity, and a 4 × 3-cm hernial orifice was identified. The hernia orifice was repaired through simple closure with sutures laparoscopically. The patient’s postoperative course was uneventful with no recurrences within the first year post-surgery.

**Conclusion:**

Combined laparoscopic and thoracoscopic surgery is a safe and effective method for Bochdalek hernial repair in adults.

## Background

Congenital posterolateral diaphragmatic hernia, also known as Bochdalek hernia, is a rare condition that primarily presents as a life-threatening cardiopulmonary disorder in the neonatal period. However, this may occasionally remain silent until adulthood, which may incidentally found by routine radiologic investigations [1–3].

Surgical repair of the hernial orifice is the definitive treatment for diaphragmatic hernia. This is warranted even in asymptomatic patients to prevent the risk of visceral herniation and strangulation. Depending on the patient’s characteristics, several approaches (e.g., laparotomy, thoracotomy, or a combination of these) may be selected. Currently, diaphragmatic hernia has been repaired through laparoscopy or thoracoscopy with or without mesh reinforcement [4, 5]. To the best of our knowledge, reports concerning combined laparoscopic and thoracoscopic surgery for diaphragmatic hernia are greatly limited. Here, we report a case of Bochdalek hernia in an adult repaired through combined laparoscopic and thoracoscopic surgery.

## Case presentation

**Fig. 1:**
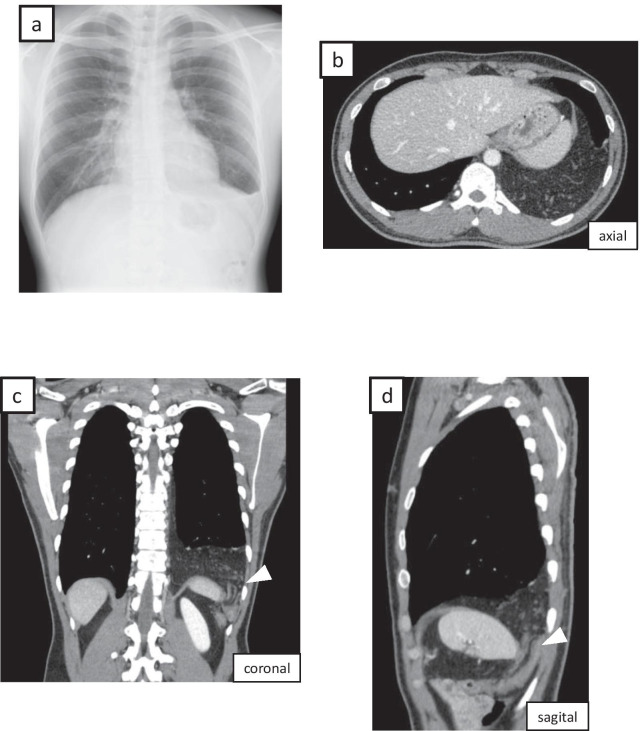
Preoperative diagnostic imaging. **a** Chest radiography showing an abnormal shadow of the left diaphragm. **b** Axial computed tomography (CT) showing herniation of omentum into the left thoracic cavity. Coronal (**c**) and sagittal (**d**) CT showing omental herniation through the orifice (arrowhead) at the posterolateral diaphragm

A 26-year-old man with unremarkable medical history presented with an abnormal shadow on routine chest radiography (Fig. 1a). Computed tomography (CT) showed herniation of a large volume of the omentum to the left thoracic cavity through an orifice in the left dorsal diaphragm (Fig. 1b–d). This was consistent with a diagnosis of Bochdalek hernia.

Despite being asymptomatic, the risk of incarceration of the prolapsed organs still exists. Hence, hernial repair was planned. A combined laparoscopic and thoracoscopic approach was preoperatively selected due to the large volume of the prolapsed omentum and the possibility of adhesion to the left thoracic cavity.

**Fig. 2:**
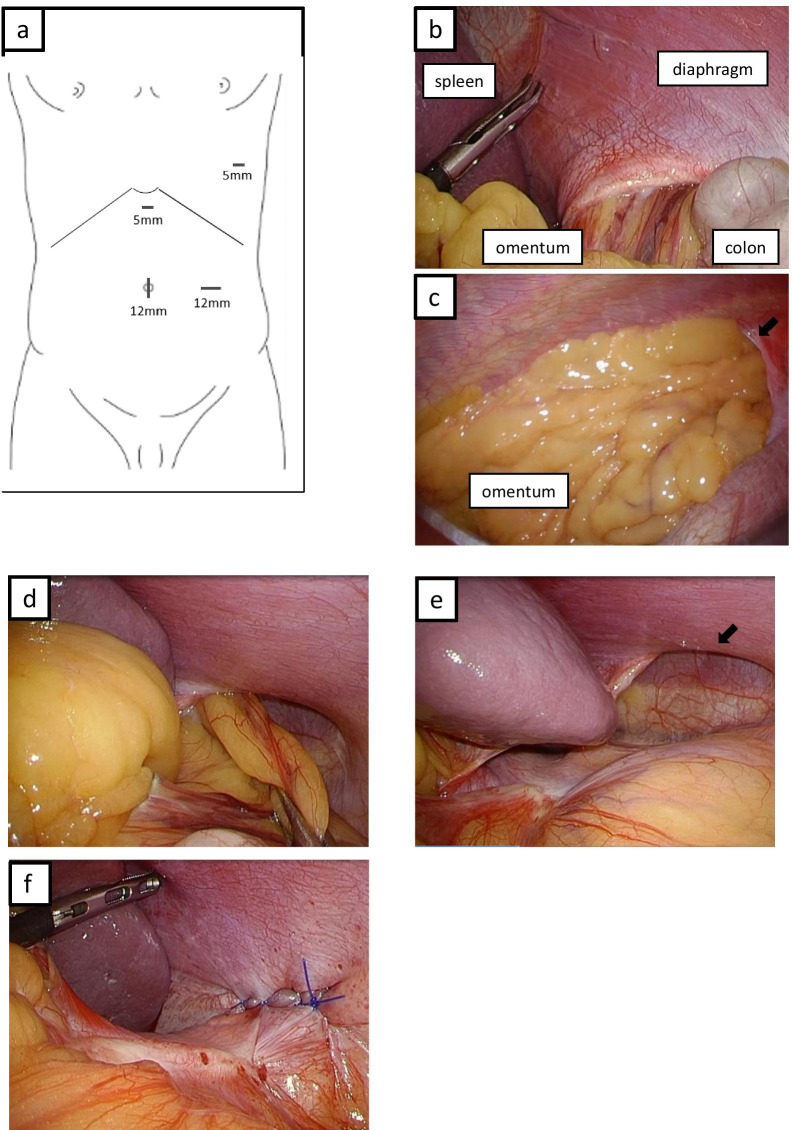
Intraoperative findings. **a** Ports placement. **b** Laparoscopy showing the orifice at the posterolateral left diaphragm with herniation of the omentum. **c** Thoracoscopy showing the hernial orifice at the posterolateral portion of the left diaphragm (arrow) and the omentum prolapsed into the thoracic cavity. **d** Drawn out omentum into the abdominal cavity. **e** A 4 × 3-cm hernia orifice (arrow). **f** Diaphragmatic orifice closed using direct sutures

During the surgery, the patient was placed in the right semi-lateral position. Four trocars were placed: (1) one 12-mm trocar at the navel, (2) one 12-mm trocar at the left abdomen, (3) one 5-mm trocar at the epigastric region, and (4) one 5-mm trocar at the left 7th intercostal space mid-axillary line (Fig. 2a). The pressure in the abdominal and thoracic cavities was maintained at 10 mmHg. On laparoscopy, the omentum prolapsed into the left thoracic cavity through the hernial orifice at the dorsolateral diaphragm (Fig. 2b). On thoracoscopy, there was no adhesion seen on the omentum in the thoracic cavity and the hernial sac (Fig. 2c).

**Fig. 3:**
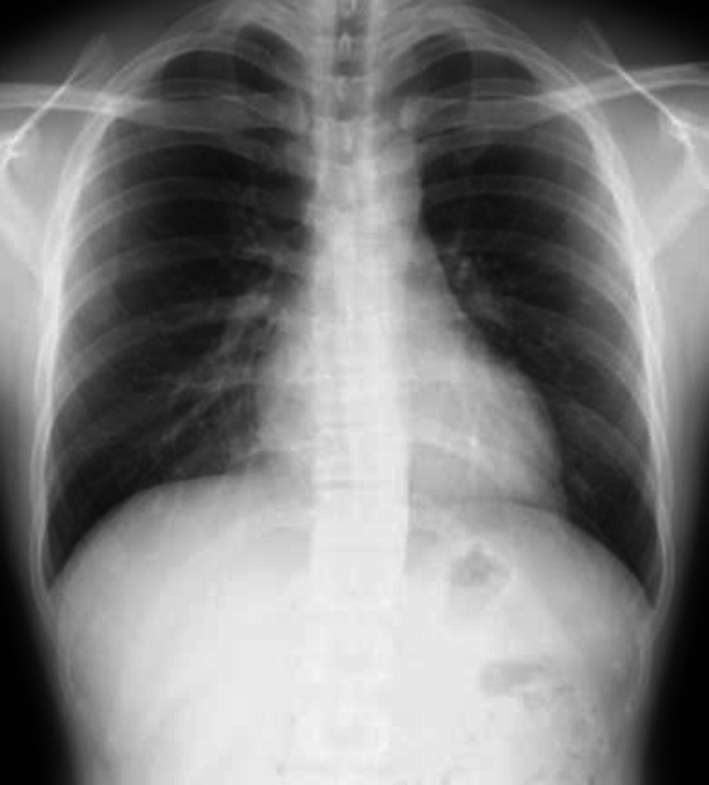
Postoperative chest radiograph 1 year after surgery

The omentum was laparoscopically drawn out from the thorax to the abdominal cavity with simultaneous observation by thoracoscopy (Fig. 2d). The hernial orifice was estimated to be 4 × 3 cm in size (Fig. 2e). After moving the omentum, the orifice was laparoscopically closed by direct sutures using 3-0 V-Loc (Covidien, Mansfield, MA, USA) and 2-0 Prolene for reinforcement (Ethicon, Somerville, NJ, USA) (Fig. 2f). The intraperitoneal pressure was reduced to 5 mmHg and there was no air leakage found from the sutured part. A 16-Fr chest drain was inserted via the left 7th intercostal space mid-axillary line. The operating time was 66 min and the total blood loss was 1 g. The postoperative course was uneventful and the patient was discharged on the 6th day after surgery. No recurrence was observed one year after the surgery (Fig. 3).

## Discussion

Vincent Alexander Bochdalek first described a congenital hernia resulting from the developmental failure of the diaphragmatic posterolateral foramina to fuse properly [6–8]. Bochdalek hernia is one of the most common abnormalities in infants with a reported incidence of approximately 1 in 3000 live births and represents approximately 80% of congenital diaphragmatic hernias [9]. This condition is usually diagnosed during infancy, which may present with signs and symptoms of cardiopulmonary insufficiency secondary to herniation of abdominal contents through the diaphragmatic defect.

In adults, Bochdalek hernia is considered extremely rare [1–3] and usually presents with vague and non-specific symptoms. The common presenting symptoms include chest pain, dyspnea, gastrointestinal bleeding, and abdominal pain [3]. In our case, he presented with none of these symptoms.

The diagnosis of diaphragmatic hernia in adults is usually established through radiologic investigations [10] such as chest radiography or barium studies. CT is the most accurate imaging modality for the diagnosis and evaluation of hernia [11, 12]. In about 80% of cases, Bochdalek hernia predominantly occurs on the left hemithorax because the right pleuroperitoneal canal closes earlier than the left [6]. In our case, the patient was initially suspected to have pleurisy based on the chest radiograph. However, due to the presence of the diaphragmatic defect on the left posterolateral area as seen in CT, a diagnosis of Bochdalek diaphragmatic hernia was made.

The conventional treatment of diaphragmatic hernia involves placing the herniated organs back to the abdominal cavity and closing the diaphragmatic defect using sutures [13, 14]. Although surgical procedures for these hernias may be performed with laparotomy, thoracotomy, or a combination of both, minimally invasive approaches (e.g., laparoscopy or thoracoscopy) have recently been approved [4, 5]. Laparoscopic surgery may allow an easier and safer return of the herniated organs back to the abdominal cavity and may provide a better examination of the whole abdominal cavity than thoracoscopic surgery [15]. In contrast, thoracoscopic surgery permits easier dissection of adhesion in the thoracic cavity than laparoscopic surgery [16]. In our case, because of the large volume of herniated omentum and the possibility of adhesion to the thoracic wall, combined laparoscopic and thoracoscopic surgery was planned to reverse the herniation and repair the orifice. Although reports of combined laparoscopic and thoracoscopic surgery exist, these were emergency operations or involved the intraoperative addition of either approach [17]. A strength of our case was that we planned combined surgery from the beginning and performed the surgery safely.

Hernial sacs have been reported to occur in 10–38% of cases [18]. In our case, no hernial sac was seen.

The hernial orifice can be closed using different methods. For cases where the defect is small, the orifice can be sutured and closed. On the other hand, for cases with large orifices (> 10 cm in diameter), mesh reinforcement may be warranted [15, 19]. As our patient’s hernial orifice was less than 10 cm in diameter, simple closure was performed with sutures. Nonetheless, there was no recurrence in the year following surgery.

## Conclusion

We have highlighted the utility of combined laparoscopic and thoracoscopic surgery for the safe and effective repair of Bochdalek hernia in adults.

## Data Availability

All data generated or analyzed during this study are included in this published article.

## Abbreviations

CT: Computed tomography
